# Challenging case of bacterial meningitis due to *Moraxella* osloensis diagnosed by 16s rRNA sequencing

**DOI:** 10.5339/qmj.2025.122

**Published:** 2025-12-15

**Authors:** Sreethish Sasi, Manal Hamed, Faiha Eltayeb, Andrez Perez-Lopez, Muna Al-Maslamani, Mohamed Abukhattab

**Affiliations:** 1 Infectious Diseases Division, Department of Medicine, Communicable Diseases Center, Hamad Medical Corporation, Doha, Qatar; 2 Division of Microbiology, Department of Laboratory Medicine and Pathology, Hamad Medical Corporation, Doha, Qatar; 3 College of Medicine, Qatar University, Doha, Qatar; 4 Microbiology Division, Department of Pathology, Sidra Medicine, Doha, Qatar; 5 Weill Cornell Medical College, Doha, Qatar *Email: Ssasi7@hamad.qa

**Keywords:** *Moraxella osloensis*, meningitis, bacterial infection, 16S rRNA sequencing, case report

## Abstract

**Introduction:**

*Moraxella* osloensis is a rare, Gram-negative, oxidase-positive coccobacillus that is infrequently identified as a pathogen in clinical diseases. It is usually opportunistic and associated with an immunocompromised host or the presence of invasive medical devices. Central nervous system infections caused by *M. osloensis* are extremely uncommon, with only a few cases documented in the literature.

**Case presentation:**

We report the case of a 49-year-old female from Cameroon with a history of diabetes mellitus and osteoarthritis who presented in Qatar with a 10-day history of headache, fever, and vomiting. Her condition progressed to confusion and persistent fever. Cerebrospinal fluid (CSF) analysis revealed purulent leukocytosis with elevated protein and glucose levels, but cultures and Gram stain were negative—findings atypical for bacterial meningitis. Empirical treatment with ceftriaxone and acyclovir was initiated. Definitive identification of *M. osloensis* was achieved via 16S rRNA sequencing of the CSF. The patient received two weeks of targeted ceftriaxone therapy, resulting in clinical improvement and full recovery by Day 17 of hospitalization.

**Discussion:**

This case highlights the diagnostic challenges associated with atypical presentations of meningitis and underscores the limitations of conventional microbiological methods in detecting rare pathogens. *M. osloensis* may not be isolated by routine culture and is frequently misidentified. Molecular diagnostic techniques, such as 16S rRNA sequencing, play a crucial role in achieving accurate identification. Given the organism’s potential for resistance and its association with invasive infections, prompt recognition and appropriate therapy are essential.

**Conclusion:**

*M. osloensis* is a rare yet potentially serious cause of bacterial meningitis, particularly in immunocompromised patients. Molecular diagnostic techniques are indispensable for confirming the pathogen in culture-negative cases. Timely diagnosis and appropriate treatment can lead to favorable outcomes, as demonstrated in this report.

## 1. INTRODUCTION

*Moraxella* osloensis is a Gram-negative, oxidase-positive, non-motile coccobacillus that is infrequently isolated from clinical samples.^1^ This bacterium is usually underreported because of its rarity and the challenges associated with its identification by phenotypic methods.^2^ It is usually opportunistic or associated with device-related infections such as those involving catheters, and it is more likely to affect immunocompromised individuals.^3–5^ The organism was first isolated and described in 1967^1,2^ and has since been reported in clinical samples from a variety of infections, including bacteremia,^4–8^ and rarely, meningitis and endocarditis.^9–11^ This is because, although *M. osloensis* is found in clinical specimens, its pathogenic potential remains largely unknown,^7^ as diseases caused by this organism are uncommon and are usually not severe. However, when such infections are invasive, they can present significant diagnostic and therapeutic challenges, often necessitating the use of advanced molecular testing methods for accurate pathogen identification. Conventional phenotypic methods often misidentify *M. osloensis* because of its non-distinctive biochemical profile and its overlap with other non-pathogenic Moraxella species. Furthermore, its slow or absent growth on routine media, particularly from sterile sites, contributes to underdetection. Commercial identification platforms (such as VITEK 2 or Analytical Profile Index [API] test strips) may either fail to identify or misclassify *M. osloensis*, necessitating the use of 16S rRNA sequencing or matrix-assisted laser desorption/ionization time-of-flight mass spectrometry (MALDI-TOF MS) for accurate species-level identification.^9^ Recent studies show that the organism is becoming more prevalent in nosocomial infections, particularly among patients with risk factors such as comorbidities or the presence of medical devices. For example, *M. osloensis* has been associated with central venous catheter infections in cancer patients,^4,5^ where accurate identification was important for guiding antimicrobial therapy. The reported cases show differences in clinical presentation and outcomes. For instance, infections have been noted in both adults and children,^4,6–8^ with clinical presentations ranging from mild respiratory symptoms to severe systemic diseases. The diverse clinical features may sometimes prevent the accurate diagnosis of this organism’s role in clinical pathology. The bacterium is sensitive to the conventional antibiotics, including penicillins and cephalosporins;^7^ however, cases of resistance have been reported, highlighting the need for appropriate antibiotic use.^4^ The treatment of infections caused by *M. osloensis* involves antimicrobial therapy guided by the isolate’s susceptibility and, when indicated, the removal of any foreign body. Thus, although *M. osloensis* is an infrequent cause of infection, it remains a potentially serious pathogen that should not be underestimated in susceptible patients.

Meningitis can be caused by bacteria, viruses, fungi, or parasites, each exhibiting distinct epidemiological characteristics. Bacterial meningitis is the most severe and often fatal form, primarily caused by *Streptococcus pneumoniae* and *Neisseria meningitidis*.^12^ Along with *Haemophilus influenzae* type b and Group B *Streptococcus*, these bacteria account for more than half of meningitis-related deaths.^12^ Viral meningitis, typically caused by enteroviruses, is more common but generally milder.^13^ Fungal meningitis (e.g., *Cryptococcus* in HIV-positive individuals) and parasitic meningitis (e.g., amoebae such as *Naegleria*) are less frequent causes.^14^ Globally, approximately 2.5 million meningitis cases occur each year, resulting in roughly 250,000 deaths.^12^ The highest incidence occurs in sub-Saharan Africa’s “meningitis belt”, which experiences frequent *N. meningitidis* epidemics, with over 20,000 cases reported in Niger since 2015.^12^ The Middle East has lower endemic rates, although outbreaks have been linked to mass gatherings such as the Hajj, prompting mandatory meningococcal vaccination.^14^

This case report aims to expand the current knowledge on this subject by describing a rare instance of partially treated bacterial meningitis caused by *M. osloensis*, highlighting the challenges in diagnosing and managing infection caused by this atypical pathogen.

## 2. CASE PRESENTATION

A 49-year-old female schoolteacher from Cameroon, visiting Qatar, initially presented to the Emergency Department at Hamad General Hospital, Doha, Qatar, with a 10-day history of headache, intermittent fever, and vomiting. The patient, with a history of diabetes mellitus and osteoarthritis, had been treated for falciparum malaria six months before this presentation. Her diabetes mellitus was controlled with oral hypoglycemic agents, with a recent HbA1c of 7.2%. At initial presentation, she was alert and oriented, with a Glasgow Coma Scale (GCS) score of 15/15 and no focal neurological deficits. Her laboratory results showed normal white blood cell (WBC), hemoglobin, and red blood cell (RBC) counts (Table 1). However, her platelet count was slightly elevated at 429×10^3^/μL, with an increased creatinine level of 133 μmol/L and a low sodium level of 130 mmol/L. Despite these findings, malaria tests were negative, and both C-reactive protein (CRP) and erythrocyte sedimentation rate (ESR) were unremarkable. The urine dipstick test was negative, and imaging—including a chest X-ray and X-rays of the hip and lumbar spine—showed severe osteoarthritis without other abnormalities. She was discharged from the Emergency Department on the same day with symptomatic treatment.

She re-presented 11 days later (referred to as Day 0 henceforth) with worsening symptoms, including a severe bilateral frontal headache, one day of confusion, and a persistent fever that had lasted two weeks. Her confusion was significant; she was disoriented in time, place, and person, with a GCS of 14/15. Despite these alarming symptoms, no neck stiffness or focal weakness was noted. Her fever peaked at 39°C in the Emergency Department. Repeated laboratory tests showed persistent abnormalities, including an elevated creatinine level of 131 μmol/L, further reduced sodium levels at 124 mmol/L, and low chloride at 87 mmol/L. A respiratory viral panel, including COVID-19, was negative. An urgent non-contrast computed tomography (CT) of the head revealed mildly prominent ventricular systems and bilateral periventricular and subcortical white matter hypodensities. An attempted lumbar puncture in the Emergency Department initially failed.

The patient was then admitted to the Internal Medicine Department for further evaluation and management. After initiating empirical treatment with intravenous ceftriaxone (2 g twice daily) and intravenous acyclovir (10 mg/kg thrice a day), cerebrospinal fluid (CSF) analysis revealed xanthochromia, an elevated WBC count of 445/μL (74% lymphocytes), and markedly increased protein and albumin levels, indicating severe inflammation. An extensive microbiological workup was performed, with CSF cultures and Gram stain yielding negative results. Polymerase chain reaction (PCR) panels for herpesviruses, enteroviruses, and arboviruses were negative. Acid-fast bacilli (AFB) stain and *Mycobacterium tuberculosis* (MTB) PCR were negative. Fungal culture and cryptococcal antigen testing were also negative. Further, imaging with magnetic resonance imaging (MRI) showed no recent infarcts but confirmed severe chronic ischemic and microangiopathic changes, along with atheromatous narrowing of the intracranial arteries. Due to the absence of a definitive diagnosis and persistent severe symptoms, the patient’s CSF sample was sent for 16S rRNA sequencing, which eventually identified *M. osloensis*, a rare cause of meningitis. This diagnosis was made after initial treatment was broadened to include empirical anti-tuberculosis therapy (ATT) and dexamethasone, owing to her deteriorating condition and a complex differential diagnosis ranging from viral encephalitis to autoimmune disorders. In classical bacterial meningitis, CSF typically shows low glucose levels due to consumption by leukocytes and bacteria. The elevated CSF glucose in our patient was likely due to her underlying diabetes mellitus, which was moderately controlled (HbA1c 7.2%). Hyperglycemia increases plasma glucose levels, and because CSF glucose is typically 60–70% of serum glucose, this may lead to relatively high CSF glucose levels. Partial treatment prior to lumbar puncture may also have altered the typical CSF findings of bacterial meningitis.

**Figure 1:**
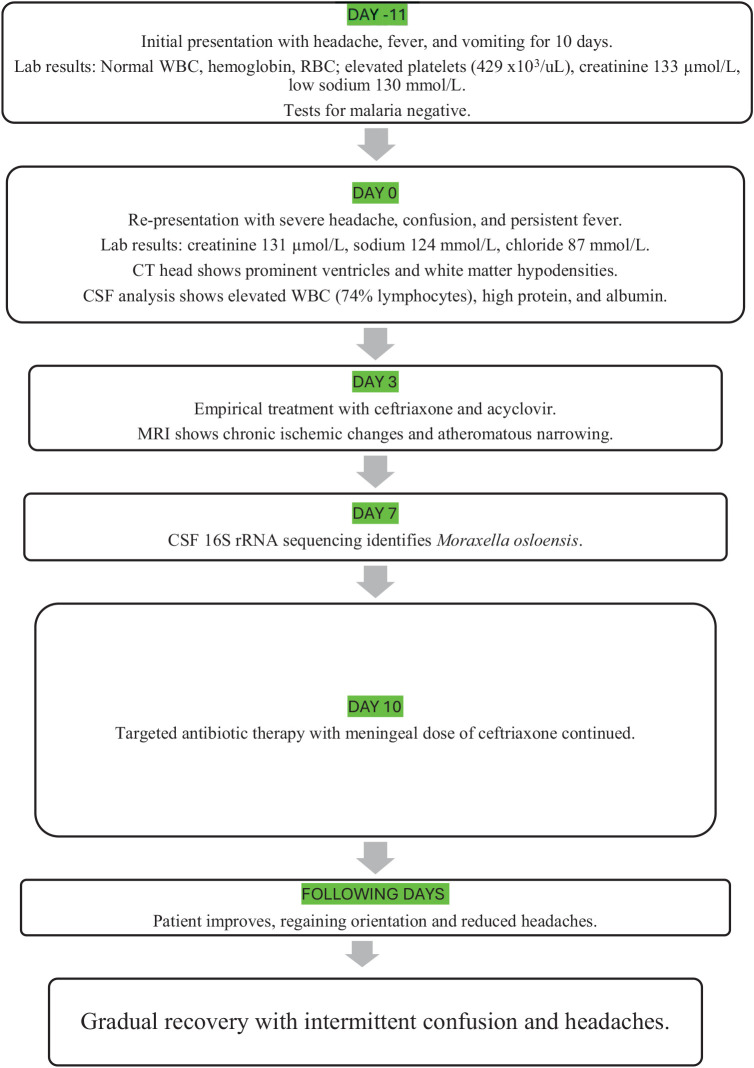
Clinical timeline of the patient’s presentation, diagnostic investigations, and therapeutic course (Day (−11 to Day +14).

The identification of *M. osloensis* prompted targeted antibiotic therapy with intravenous ceftriaxone (2 g twice daily) for 2 weeks, resulting in gradual improvement of the patient’s condition. Acyclovir, ATT, and dexamethasone were discontinued immediately. By Day 10, she was oriented and alert, and over the following days, she showed slow but continuous recovery, with intermittent confusion and headaches, yet overall improvement in cognitive and physical functions. By Day 17 of hospitalization, the patient had returned to her clinical baseline and was discharged. No additional medications were prescribed, apart from her usual oral hypoglycemics for diabetes. She opted to return to her home country after discharge; therefore, outpatient follow-up was not feasible. A timeline of events is presented in Figure 1.

This case underscores the challenges of diagnosing atypical bacterial meningitis, especially when initial presentations are complicated by partial treatments and non-specific symptoms, and highlights the crucial role of advanced molecular techniques, such as 16S rRNA sequencing, in identifying rare pathogens.

## 3. DISCUSSION

The identification and management of infections caused by *M. osloensis* present unique clinical challenges, as highlighted in several case reports and reviews in the literature. This discussion synthesizes findings from various sources to provide a comprehensive overview of the complexities associated with *M. osloensis* infections, particularly in comparison with more commonly encountered pathogens. *M. osloensis* is an uncommon pathogen, often isolated from patients with underlying health conditions or those undergoing invasive procedures.^3–5^ Although the organism is part of the normal human respiratory tract flora, it can cause invasive infections under certain conditions. The cases reviewed indicate that this organism can cause a range of clinical manifestations, from bacteremia and pneumonia to rarer conditions, such as meningitis and catheter-related infections.^4,7^ For instance, Hadano et al.^5^ described a case of a central venous catheter infection in a cancer patient, highlighting the organism’s ability to cause significant morbidity in immunocompromised individuals. Similarly, other reports have documented its association with respiratory tract infections and bacteremia, particularly in patients with predisposing factors such as malignancies, chronic diseases, and indwelling devices.^3–6^ One of the major challenges in managing infections caused by *M. osloensis* is its identification. Conventional biochemical tests often fail to accurately identify this organism because of its phenotypic similarities to other Moraxella species.^9^ Advanced diagnostic approaches, such as MALDI-TOF MS, 16S rRNA sequencing, and metagenomic next-generation sequencing (mNGS) have become valuable tools when routine microbiology fails.^9,15^ 16S rRNA sequencing targets the conserved 16S rRNA gene to identify bacteria, even after prior antibiotic therapy, and is often employed as the next step in cases of culture-negative meningitis.^15^ Building on this, 16S-based mNGS employs high-throughput sequencing to detect multiple or fastidious organisms with greater sensitivity, thereby expanding diagnostic yield.^15^ These methods are particularly important in culture-negative cases, where prior treatment or fastidious pathogens can obscure diagnosis. They have identified not only classical meningitis pathogens, such as *Streptococcus pneumoniae* and *Listeria monocytogenes*, but also fastidious or atypical bacteria, including *Brucella, Nocardia*, and *Actinomyces*.^15^ By identifying pathogens missed by standard cultures, these advanced methods facilitate timely, targeted therapy and reduce reliance on prolonged empirical treatment.^15^

The challenges in diagnosing *M. osloensis* infections may contribute to underreporting and, consequently, a limited understanding of its epidemiology and clinical significance. Therefore, enhancing laboratory capabilities to incorporate molecular diagnostic tests is crucial for improving the detection and characterization of this and similar pathogens. In this case, the exact source of the *M. osloensis* infection could not be definitively determined. However, the most likely source is endogenous colonization of the upper respiratory or gastrointestinal tract, followed by hematogenous spread. The patient had no indwelling devices, but her comorbid diabetes mellitus may have predisposed her to mucosal barrier disruption and transient bacteremia. It is possible that unrecognized upper airway colonization or subclinical sinusitis, as described by Fox-Lewis et al.^10^ contributed to this case.

Ceftriaxone was chosen based on empirical treatment guidelines for community-acquired meningitis and supported by literature demonstrating *M. osloensis* susceptibility to β-lactams and cephalosporins.^4,7^ Although empirical therapy with ceftriaxone aligned with standard recommendations for community-acquired meningitis, the patient’s persistent symptoms and atypical CSF profile raised concern for alternative diagnoses, including tuberculosis and viral encephalitis. In this context, molecular confirmation was not only diagnostic but also reassured clinicians that continuing β-lactam therapy alone was sufficient, thereby preventing unnecessary broadening or prolongation of treatment. No susceptibility testing was performed because no isolate was cultured; this represents a limitation, and the treatment was guided by published susceptibility profiles. The literature indicates that *M. osloensis* is generally susceptible to a broad spectrum of antibiotics, including penicillins and cephalosporins. However, treatment may be complicated by the organism’s variable susceptibility patterns and the specific clinical context of the infection. Effective management often requires not only the administration of appropriate antibiotics but also, in cases such as catheter-related infections, the removal of the infected device. In the cases reviewed, outcomes varied significantly depending on the infection site, the patient’s overall health status, and the timeliness of both diagnosis and intervention. For example, the patient reported by Hadano et al.^5^ experienced a favorable outcome despite the severity of the underlying condition and the potential complications associated with catheter-related infections. This highlights the importance of a tailored treatment approach that considers both the microbiological and clinical aspects of each case. In comparison, infections caused by the more commonly encountered organisms, such as *Staphylococcus aureus* or *Pseudomonas aeruginosa*, are generally associated with more straightforward diagnostic and management protocols, owing to the extensive body of knowledge regarding their pathogenic mechanisms, clinical presentations, and treatment strategies. In contrast, the rarity and relative obscurity of *M. osloensis* contribute to the increased complexity and higher risk of initial mismanagement observed in cases involving this organism. The growing recognition of *M. osloensis* as a potential pathogen in a variety of clinical scenarios highlights the need for heightened awareness among clinicians and microbiologists. It also emphasizes the importance of including this organism in the differential diagnosis of infections, particularly in immunocompromised patients or those with indwelling medical devices. This case highlights the importance of clinical vigilance in patients with underlying comorbidities, such as diabetes mellitus, as atypical pathogens may present with subtle or confounding features. Broader implementation of molecular diagnostic platforms in low-resource settings is essential for detecting rare pathogens and initiating timely, targeted therapy. A summary of published cases of *M. osloensis* infections is presented in Table 2.

## 4. CONCLUSION

This case report highlights the diagnostic and therapeutic challenges posed by the rare pathogen *M. osloensis*, which caused partially treated bacterial meningitis in a 49-year-old patient. Although part of the normal respiratory flora, its pathogenic potential was revealed through advanced 16S rRNA sequencing, highlighting the importance of considering such uncommon pathogens in atypical presentations of meningitis. The successful resolution of this case with targeted antibiotic therapy, following initial diagnostic uncertainty, emphasizes the critical role of precise microbial identification in effective infection management. This case serves as an important reminder of the need for heightened clinical awareness and the use of molecular diagnostics in the treatment of elusive infectious diseases. In conclusion, although *M. osloensis* is an uncommon and often overlooked pathogen, its ability to cause significant clinical infections underscores the need for a better understanding of its microbiological characteristics, potential resistance patterns, and effective treatment strategies. The ongoing development and application of advanced diagnostic techniques will be pivotal in achieving these objectives, thereby improving patient outcomes in infections caused by this challenging bacterium.

## AUTHORS’ CONTRIBUTION

All authors made a significant contribution to the work reported—whether in the conception, study design, execution, acquisition of data, analysis and interpretation, or in all these areas. All authors took part in drafting, revising, or critically reviewing the article; approved the final version to be published; agreed on the journal to which the article has been submitted; and accepted responsibility for all aspects of the work.

## ETHICS STATEMENT

This study was conducted in accordance with the principles of the Declaration of Helsinki and was approved by the Institutional Review Board (IRB) of the Medical Research Center (MRC), Hamad Medical Corporation (HMC), Qatar (MRC-04-24-247). Written informed consent was obtained from the patient’s immediate relative for the publication of the case information.

## ACKNOWLEDGMENTS

The authors acknowledge the support of the Infectious Diseases and Microbiology divisions of Hamad Medical Corporation, the Microbiology and Genomics divisions of Weill Cornell Medicine, Qatar, and the Medical Research Center (MRC) of Hamad Medical Corporation.

## FUNDING

The research was supported by the Medical Research Center (MRC) of Hamad Medical Corporation (HMC), Qatar, under grant number MRC-04-24-247.

## COMPETING INTEREST

The authors have no conflicts of interest to declare.

## Figures and Tables

**Table 1.** Clinical and laboratory findings in the Moraxella osloensis meningitis case.

Parameter	Normal range	Day 0	Day 5	Day of discharge
**Blood**				
Sodium (mmol/L)	133–146	124 (Low)	126 (Low)	132 (Borderline)
Potassium (mmol/L)	3.5–5.3	4.4 (Normal)	4.8 (Normal)	4.2 (Normal)
Chloride (mmol/L)	95–108	87 (Low)	88 (Low)	93 (Low)
Creatinine (μmol/L)	44–80	131 (High)	118 (High)	110 (High)
CRP (mg/L)	0.0–5.0	6.1 (High)	4.9 (Normal)	3.2 (Normal)
**Cerebrospinal fluid (CSF)**				
CSF WBC (cells/μL)	0–5	445 (High)	417 (High)	Normal
CSF neutrophil (%)	0–6	24 (High)	14 (High)	Normal
CSF protein (g/L)	0.15–0.45	4.28 (High)	3.01 (High)	Normal
CSF glucose (mmol/L)	2.22–3.89	4.39 (High)	3.84	Normal
CSF lymphocyte (%)	40–80	74	83	Normal
CSF monocyte (%)	15–45	2 (Low)	3 (Low)	Normal
CSF RBC (cells/μL)	0	10	5	Normal

**Table 2.** Summary of published clinical cases of Moraxella osloensis infection.

S.no.	Author	No. of cases	Clinical scenario	Patient age/sex	Underlying conditions	Diagnosis method	Treatment	Outcome
1	Hadano et al.^5^	1	Central venous catheter-related bacteremia in a cancer patient	67-year-old male	Pancreatic cancer with liver metastases	16S rRNA sequencing	Cefepime for 14 days	Complete recovery
2	Koleri et al.^7^	9	Bacteremia of varying clinical significance (2 significant, 7 contamination)	Median age 47 years (range 39–80) 6 males, 3 females	Diabetes, malignancies, immunosuppression	Blood culture and MALDI-TOF MS	Ampicillin, ceftriaxone	Complete recovery
3	Tabbuso et al.^8^	1	Pediatric bacteremia and meningitis cases	2-month-old female	None (in pediatric case)	Blood culture, 16S rRNA sequencing	Cefotaxime	Complete recovery
4	Roh et al.^9^	3	Meningitis in 2 children and an adult cancer patient	4, 15, and 81 years (2 males and 1 female)	Pancreatic cancer, liver cirrhosis (1 case)	CSF culture, 16S rRNA sequencing	Cefotaxime, ampicillin, ceftazidime, netilmicin	Complete recovery
5	Fox-Lewis et al.^10^	1	Meningitis due to subclinical sinusitis with bony erosion	31-year-old male	Subclinical sinusitis	CSF PCR for 16S rDNA	Chloramphenicol, doxycycline	Complete recovery
6	Li et al.^11^	1	Meningitis with diplopia and intracranial pressure	18-year-old male	None	mNGS	Cefoperazone/sulbactam	Complete recovery
7	This case	1	Meningitis	49-year-old female	Diabetes, osteoarthritis	16S rRNA sequencing	Ceftriaxone	Complete recovery
